# Evaluating the efficacy and timing of blood purification modalities in early-stage hyperlipidemic acute pancreatitis treatment

**DOI:** 10.1186/s12944-023-01968-z

**Published:** 2023-11-29

**Authors:** Jianjun Wang, Yang Xia, Yuan Cao, Xianfu Cai, Shichun Jiang, Yougang Liao, Mingsong Shi, Huiwen Luo, Decai Wang

**Affiliations:** 1 Department of Hepatobiliary Surgery, Mianyang Central Hospital, School of Medicine, University of Electronic Science and Technology of China, Mianyang, 621000 China; 2 Department of Neurosurgery, Mianyang Central Hospital, School of Medicine, University of Electronic Science and Technology of China, Mianyang, 621000 China; 3 Department of Urology, Mianyang Central Hospital, School of Medicine, University of Electronic Science and Technology of China, Mianyang, 621000 China; 4 Nuclear Medicine Laboratory, Mianyang Central Hospital, School of Medicine, University of Electronic Science and Technology of China, Mianyang, 621000 China

**Keywords:** Hypertriglyceridemia, Triglycerides, Acute pancreatitis, Blood purification, Therapy

## Abstract

Hypertriglyceridemia-induced acute pancreatitis (HTG-AP) is characterized by a violent cytokine storm-driven inflammation and is associated with a predisposition to severe disease. The treatment strategy for HTG-AP consists mainly of conventional symptomatic and lipid-lowering treatments. For early-stage HTG-AP, blood purification (BP) can rapidly and effectively reduce serum triglyceride and inflammatory cytokine levels, block the development of systemic inflammatory response syndrome, and improve patient outcomes. Currently, the primary modalities for BP in patients with HTG-AP include plasma exchange, hemoperfusion, and hemofiltration. When using BP to treat patients with HTG-AP, a comprehensive analysis incorporating the elevated lipid levels and severity of the patient’s condition contributes to the selection of different treatment modes. Moreover, the timing of the treatment is also imperative. Early intervention is associated with a better prognosis for patients with HTG-AP requiring lipid-lowering treatment.

## Introduction

Speck et al. first reported a connection between hypertriglyceridemia(HTG) and acute pancreatitis (AP) in 1865 [1]. Due to changes in living standards and dietary patterns, the incidence of obesity-related dyslipidemia has increased significantly over the years [2], resulting in a year-on-year increase in hypertriglyceridemia-induced acute pancreatitis (HTG-AP) cases worldwide [3]. In Western countries, HTG is the most common cause of AP after cholestasis, gallstones, and alcohol consumption [4–6], and the incidence of AP induced by alcohol plus a high-fat diet is higher than that of idiopathic AP [5]. HTG is also the second most significant cause of AP after cholelithiasis in China [7], surpassing the prevalence reported by international registries [8], which may be attributed to changes in dietary habits and genetic factors. Epidemiological studies have shown that the prevalence of HTG-AP has increased to 14% and appears to increase yearly [9, 10]. Pathophysiological mechanisms underlying HTG-AP are complex and involve multiple factors [11, 12]. Many studies have suggested that the HTG level is a significant risk factor for HTG-AP development [3, 13]. Qiu et al. reported that the risk of HTG-AP in patients with moderate-to-severe HTG was markedly increased compared with patients with mild HTG [10]. Qiu et al. also reported a positive association between serum triglyceride (TG) levels and AP risk. Studies have demonstrated that the risk of AP increases by 4% for each 1.13 mmol/L rise in serum TG [14]. The risk of AP was 5% when the serum TG > 11.3 mmol/L and climbed to 10–20% when the serum TG > 22.6 mmol/L [9, 10], and there was an observed trend toward a younger age [3, 15–17], with a higher prevalence in males and a tendency to be associated with other risk factors such as diabetes, fatty liver, and dyslipidemia [12, 15, 18]. In female patients, HTG-AP occurs primarily during pregnancy and is associated with familial HTG, with an incidence of approximately 1 in 25,000 [19]. Additionally, the recurrence rate of HTG-AP was significantly higher than that of AP of other etiologies [10, 20]. Elevated TG levels in HTG-AP lead to more free fatty acids released during pancreatic lipase hydrolysis than in AP induced by other causes [21], contributing to the development of systemic inflammatory response syndrome (SIRS) and progression to hyperlipidemic severe acute pancreatitis (HLSAP) [10, 22], with more severe pancreatic necrosis later in the course of the disease.

Based on the studies published so far, this review article aimed to analyze how elevated lipid levels and the severity of the patient’s condition influences the choice of different treatment modes. We compared and contrasted the advantages and disadvantages of various modes, such as lifestyle modification, pharmacotherapy, and surgery, and provided evidence-based recommendations for clinical practice.

## Diagnosis

Various studies have regarded TG > 11.3 mmol/L as a diagnostic criterion for severe HTG, indicating that the incidence rate of HTG-AP is 10–20% [12]. In a systematic review exploring the etiology and severity of HTG-AP, Rosalie et al. reported that TG > 11.3 mmol/L was a crucial factor for the development of AP [23]. However, Sandhu et al. reported that even at TG < 20.0 mmol/L, there were no cases of HTG-induced AP in their included studies [24]. These findings suggest individual variations among patients, although the risk of HTG-AP onset is remarkably increased at TG > 11.3 mmol/L. Other studies have revealed different standards. Individuals whose serum TG is between 5.65 and 11.3 mmol/L and those with fasting venous chylous blood simultaneously fulfil the diagnosis criteria for HTG-AP [10, 25]. The diagnosis of HTG-AP first requires a precise diagnosis of AP based on the guidelines reported in the Revised Atlanta Classification of Acute Pancreatitis [26]. The guidelines are as follows: (i) The characteristics of abdominal pain are consistent with AP: sudden onset of acute, persistent, and severe epigastric abdominal pain with radiating pain to the back and shoulder. (ii) Laboratory test results are consistent with AP: serum amylase or lipase levels not less than three times the upper limit of the reference range. (iii) Abdominal imaging shows changes following the imaging features of AP: pancreatic edema or peripancreatic exudate. According to the guidelines, two criteria must be fulfilled to confirm the diagnosis.

**Scheme 1:**
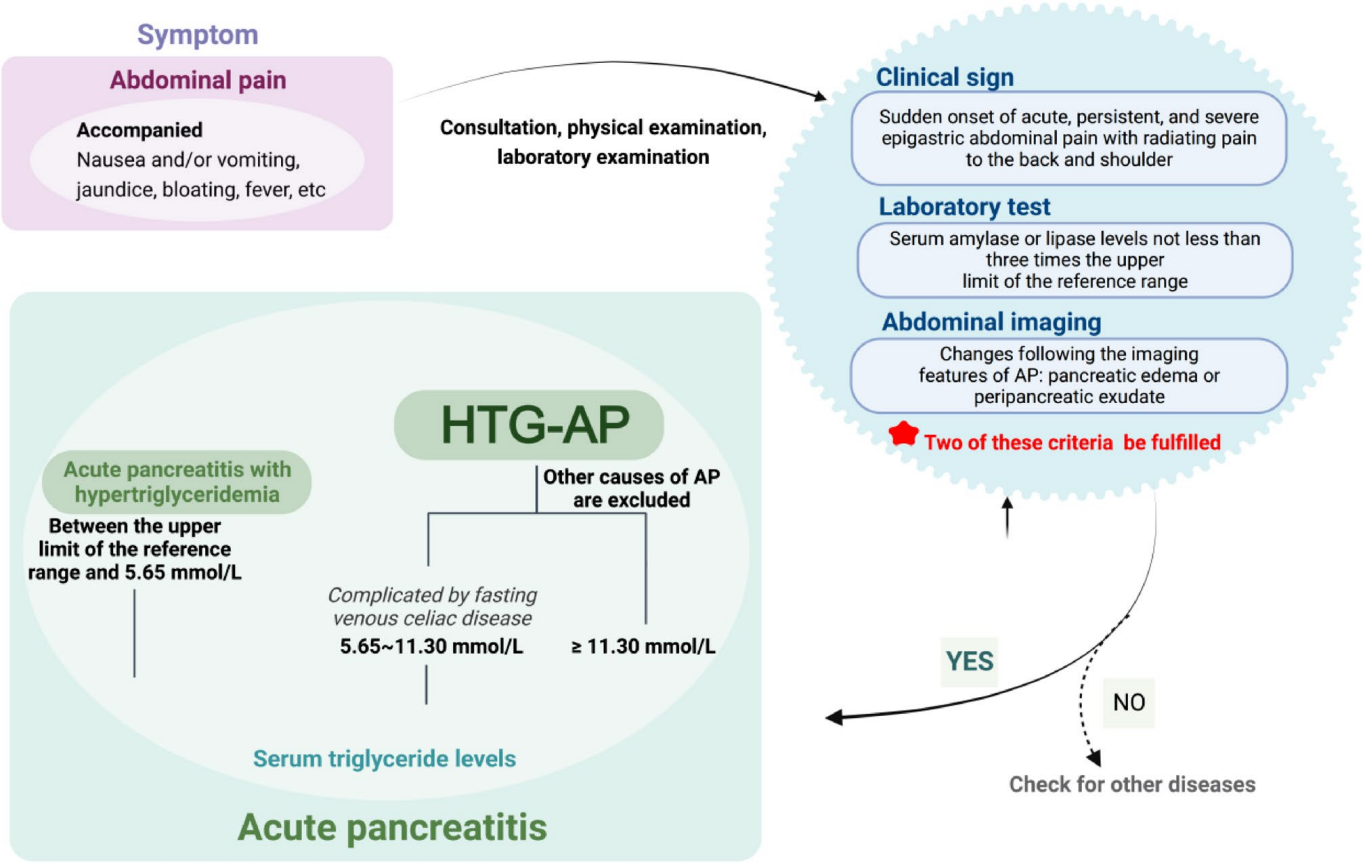
A schematic diagram of diagnosis for HTG-AP

The diagnosis of HTG-AP can be made by applying the AP criteria combined with the following diagnostic standards: (i) Serum TG > 11.3 mmol/L while excluding other causes of AP. (ii) Fasting serum TG between 5.65 and 11.3 mmol/L, complicated by fasting venous chylous blood, to rule out other causes of AP. (iii) The threshold of serum TG levels that leads to HTG-AP varies individually, as there is a positive correlation between TG levels and the risk of AP. Therefore, it is crucial to consider the possibility of HTG-AP early on when serum TG levels significantly exceed normal physiological levels and other causes of AP have been excluded (Scheme 1).

## Treatment

### Conventional treatment

Conventional treatments for symptoms include fluid resuscitation, analgesia, inhibition of gastric acid and pancreatic enzyme secretion, and other modalities.

Early fluid resuscitation is fundamental to AP treatment. It is vital to prevent multiple organ dysfunction syndrome(MODS) and SIRS by improving microcirculatory deficits and controlling inflammatory responses. Therefore, fluid resuscitation should be performed within the first 24 h of symptom onset [27]. Currently, rehydration fluids are available as crystalloids and colloids; however, there are no clear recommendations concerning their dosage and proportions. In addition, studies have shown that lactated Ringer’s solution (LR) is superior to normal saline (NS) for controlling SIRS in crystal therapy [28]. In contrast, NS is the preferred choice for patients with hypercalcemia because of the calcium content of LR [29].

It is also necessary to reduce the workload on the pancreas. Proton pump inhibitors (PPI) and fasting indirectly inhibit pancreatic secretion by reducing gastric acid secretion. Growth hormone and its analogues (octreotides) directly inhibit pancreatic exocrine secretion. Protease inhibitors inhibit the release and activity of pancreatic proteases and reduce pancreatic injury [30]. Meng et al. reported that combining ustekin and octreotide reduced endothelin and endotoxin levels and improved immune function in patients with AP [30].

Furthermore, in patients with HTG-AP, nutritional support requires special attention to control lipid intake, aiming to prevent a suboptimal outcome due to elevated serum TG [31, 32]. Analgesic modalities such as NSAIDs, opioids, and epidural analgesia may help relieve upper abdominal pain and radiating back pain [33]. However, the prophylactic use of antibiotics in AP remains controversial. Reports have suggested that prophylactic antibiotics fail to reduce the rates of pancreatic or peripancreatic infections, surgery, or death in patients with severe acute pancreatitis (SAP) [31, 34, 35].

### Etiological treatment

Apart from conventional treatments for AP, HTG-AP treatment requires consideration of lipid-lowering therapies. After establishing that TG levels were associated with the development of organ failure in HTG-AP patients, studies focused on whether the rate of triglyceride decline was related to improved clinical outcomes, although no conclusions have been drawn [36]. The key to treatment lies in rapid and effective clearance of serum TG and inflammatory mediators in the shortest time to relieve or even block SIRS and effectively prevent the progression of HTG-AP. According to available studies, the reduction and limitation of serum TG to less than 5.65 mmol/L positively halts the progression of HTG-AP [33–39]. Lacking high-quality evidence due to most of the available evidence at this stage being retrospective, single-centre, small sample size, there is no specific triglyceride-lowering therapy for HTG-AP patients, and the lipid-lowering treatments available at this stage are mainly drugs and BP.

Fibrate is the drug of choice for lipid-lowering treatment. It reduces TG levels by stimulating low-density lipoprotein (LPL) release. Combining fibrates and statins can significantly reduce lipid levels in patients with severe HTG that fibrates alone cannot control [40]. However, this combination treatment can increase the risk of causing myopathy and hepatotoxicity. In addition to commonly used drugs, omega-3 fatty acids can also help decrease TG levels [41].

Insulin increases the degradation of celiac particles and TG by stimulating LPL activity. Some studies have shown that insulin can reduce serum TG levels by 50–75% within three days [37, 42]. Clinically, intravenous infusion facilitates precise control of insulin intake and adding 5% dextrose to the infusion set is desirable to prevent hypoglycemic side effects [43, 44]. Heparin reportedly induces the release and attachment of LPL to endothelial cells and decreases serum TG levels [45]. Low-molecular-weight heparin is clinically recommended for patients with HTG-AP without bleeding tendencies. However, long-term treatment with heparin may lead to the accumulation of circulating celiac particles and the rebound of TG levels due to the depletion of LPL on the endothelial cell surface [46]. Moreover, heparin may increase the risk of bleeding from pancreatic vascular beds [47]. Therefore, the decision to use heparin alone in treating HTG-AP and its exact dose and duration still need to be weighed against its advantages and disadvantages.

**Scheme 2:**
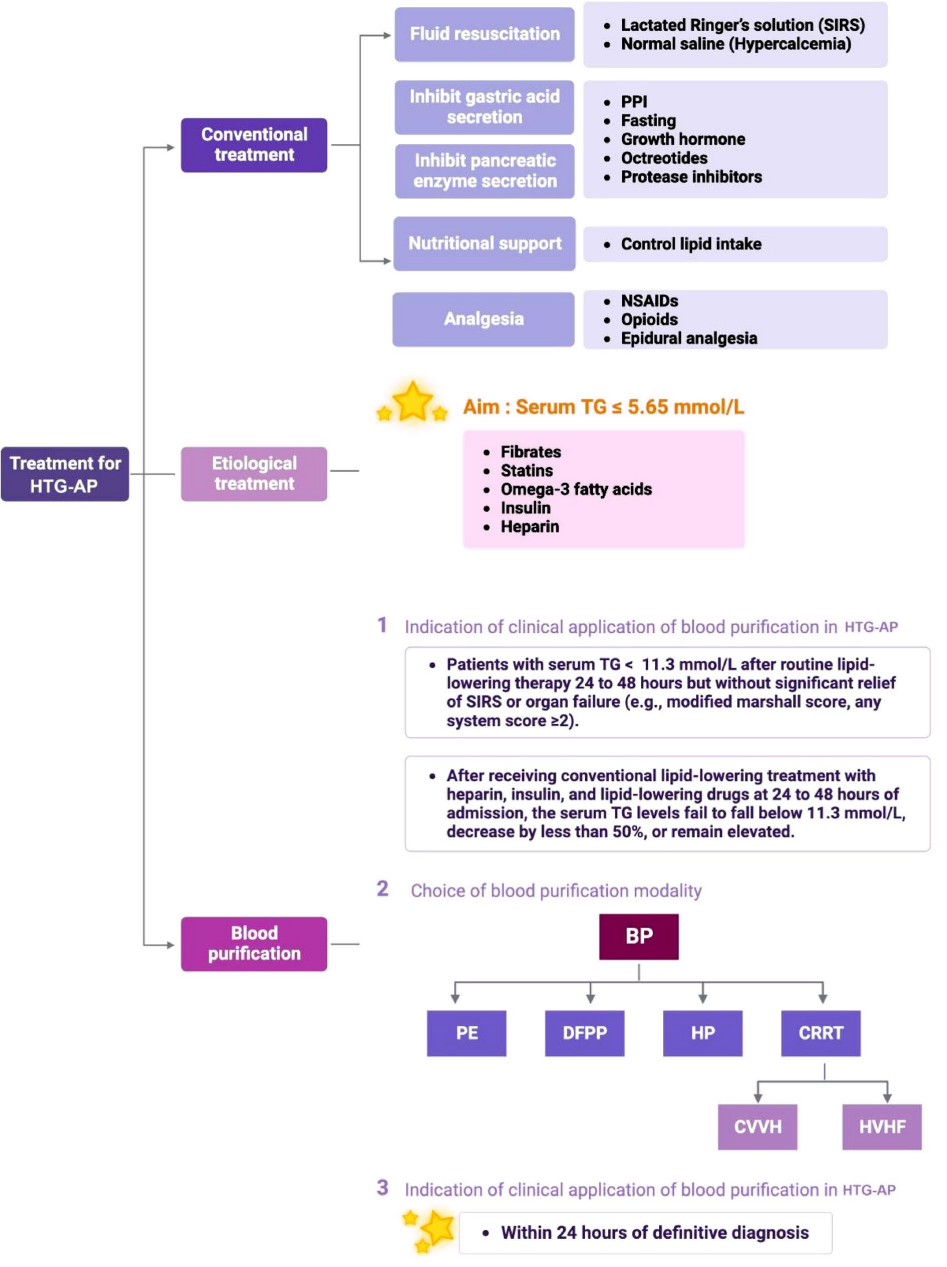
The schematic diagram of treatment for HTG-AP

A heparin and insulin combination can be used as first-line treatment for severe HTG-AP [48]. This combination strategy was successfully used in four patients with HTG-AP, resulting in a 75% decrease in serum TG levels after three days and a significant improvement in clinical symptoms [48]. These findings highlight the potential of heparin and insulin as an effective therapeutic approach for managing severe HTG-AP. Future research should further explore the efficacy and safety of this treatment modality in larger cohorts to establish its place in clinical practice and identify any potential limitations or side effects. Additionally, investigating the optimal dosing regimens and treatment duration may provide valuable insights to optimize patient outcomes in managing HTG-AP (Scheme 2).

## Indication of clinical application of blood purification in HTG-AP

In contrast to the slow onset and suboptimal effect of drugs, blood purification (BP) can help achieve the therapeutic need of rapid reduction of serum TG levels within a short duration and is regarded as a more effective and common treatment for HTG-AP in clinical practice. There is no definitive indication for BP in HTG-AP treatment. Several studies have reported that serum TG > 11.3 mmol/L could be rapidly reduced to below 11.3 mmol/L using BP treatment, which notably prolongs the survival time [49]. In addition, Chang et al. highlighted that when serum TG was > 56.5 mmol/L, which is referred to as HLSAP, treatment with double filtration plasma exchange significantly shortened the length of hospital stay and readmission rate [50]. In 2017, two patients experienced re-elevation in serum TG levels after a short-term reduction following conventional treatment with fluid resuscitation, pain management, insulin, and lipid-lowering medication. One patient received plasma exchange treatment on the fourth day of admission, and the serum TG level decreased by 57.8% after three treatments. Another patient received two plasma exchange treatments on the third and fourth day, with a 60.6% decrease in serum TG levels [51]. Studies have suggested that patients with HLSAP and APACHE II scores ≥ 8, organ dysfunction, or Balthazar grade D/E should immediately be transferred to the intensive care unit and receive non-pharmacological treatment if conventional treatment fails. Non-pharmacological treatments are recommended in such cases, with albumin being the primary replacement solution and the most common treatment modality for this condition [38].

Based on the findings from the above studies, patients with HTG-AP are considered for BP treatment when they meet the following criteria: (i) If the serum TG levels fail to fall below 11.3 mmol/L, decrease by less than 50%, or remain elevated after receiving conventional lipid-lowering treatment with heparin, insulin, and lipid-lowering drugs at 24 to 48 h of admission, this may be an indication for BP treatment. (ii) For patients with serum TG < 11.3 mmol/L after routine lipid-lowering therapy 24 to 48 h but without significant relief of SIRS or organ failure (e.g., Modified Marshall Score, any system score ≥ 2), early consideration of BP therapy is recommended.

Early identification of high-risk patients and timely initiation of intensive care measures, including the appropriate use of albumin, can significantly improve patient outcomes in HLSAP. Further research should continue to explore and refine the best practices for managing HLSAP in the intensive care setting to optimize treatment efficacy and patient prognosis.

## Choice of blood purification modality

BP involves drawing blood out of the body and using blood cell separators, blood filters, and other specific equipment to eliminate and replace disease-causing substances, which can reduce organ damage and cure diseases.

### Plasma exchange (PE)

During PE, a blood cell separator isolates plasma from blood cells and subsequently removes serum TG from the plasma. During this process, the organic fraction of blood is simultaneously returned to the patient and replaced with an equal volume of replacement fluid. PE can quickly remove the accumulated chylomicrons in the blood circulation during a short period and prevent the release of inflammatory mediators to balance the pro-inflammatory and anti-inflammatory responses in patients. These measures effectively relieve SIRS and improve vital organ function, preventing further deterioration of the patient’s condition. Freshly frozen plasma and albumin are commonly used as replacement solutions for PE [52]. Fresh frozen plasma as a replacement solution can rapidly eliminate TG and replenish the lipoprotein enzymes and lipids required for TG decomposition [53]. However, it should be noted that PE requires the transfusion of large amounts of allogeneic plasma, which can lead to fever, allergic reactions, and other transfusion-related complications [54]. Using albumin as a replacement fluid can offer the following advantages [55]: (i) It can help avoid fever and allergic reactions caused by fresh frozen plasma replacement. This reduces the risk of transmission of transfusion-associated diseases. (ii) It does not require consideration of blood type matching. (iii) The medical costs associated with transfusion are lower. However, it should also be noted that the lack of ApoCII and LPL in the albumin replacement fluid leads to celiac disease, which increases serum TG levels and promotes AP progression [56, 57].

Currently, in clinical practice, PE mainly employs albumin as the replacement fluid [37]; however, there is a lack of conclusive evidence to recommend a specific PE fluid (albumin vs. fresh frozen plasma). Owing to the potential risk of membrane plasmapheresis blockage, researchers recommend centrifugal plasmapheresis because of its safety and efficacy [37]. Five case reports from 2005 showed notable clinical improvements in patients diagnosed with HTG-AP after receiving PE within 48 h of admission. All patients experienced significant relief from abdominal pain and serum TG reduction within 30 min of treatment. Three of these patients achieved near-normal TG levels after one treatment, and two others achieved the treatment goal after two or three treatments [58]. A retrospective study showed that PE effectively reduced TG concentrations in the acute phase of HTG-AP, relieved clinical manifestations, such as abdominal pain, and alleviated early symptoms as soon as possible. However, it did not significantly reduce morbidity or mortality [36]. Therefore, many previous studies have suggested that PE is the preferred BP method to effectively reduce serum TG concentration in HTG-AP patients with serum TG > 11.3 mmol/L. However, the choice of BP-lowering therapy for triglycerides remains controversial, as a large, multicenter prospective cohort study in 2023 in patients with HTG-AP showed no incidence or duration of organ failure, but was associated with increased ICU demand [59].

### Double filtration plasmapheresis (DFPP)

Recently, HTG-AP lipid-lowering therapy has recommended DFPP. After the blood flows through the membrane plasma separator, the separated plasma flows through the plasma-component separator with a smaller membrane pore size. During DFPP, plasma-component separators with different pore sizes can selectively remove plasma proteins [60]. This process facilitates the isolation of other pathogenic factors with relative molecular masses larger than albumin [61], based on which plasma components containing undiscarded albumin are subsequently redelivered to the patient. In contrast to PE, the characteristics of DFPP have positive implications for reducing blood product-dependent infectious diseases. In addition, DFPP can effectively remove cytokines that promote HTG-AP progression, such as oxidatively modified ApoCs [62]. As it does not require additional allogeneic plasma supplementation [63], DFPP is considered a preferable option for blood-shortage situations. It can help prevent complications associated with high-dose allogeneic plasma transfusions while reducing plasma consumption [64]. However, DFPP treatment can decrease fibrinogen and albumin levels in patients with HTG-AP [65], resulting in immune function dysfunction.

Studies have reported that DFPP is more efficient in clearing TG than PE during HTG-AP treatment. A 2020 controlled study reported that when serum TG > 11.3 mmol/L, early implementation of DFPP was more rapid and effective in reducing serum TG levels than in the control group [66]. Moreover, in patients with HLSAP with serum TG > 56.5 mmol/L, DFPP rapidly reduced serum TG levels, eliminated inflammatory mediators, and shortened the length of hospital stay. Prophylactic DFPP treatment can help prevent the recurrence of HTG-AP in patients with a history of HLSAP [50, 67]. However, reports regarding DFPP treatment are currently relatively sparse, and treatment is not yet available in many medical centers. Therefore, for patients with HLSAP with a serum TG > 56.5 mmol/L, we recommend DFPP as the priority treatment if available.

### Hemoperfusion (HP)

HP uses the adsorption effect of a solid sorbent to remove recalcitrant substances that are difficult to remove, including drugs, specific toxins, lipids, and other metabolites, via hemodialysis. Currently, HTG-AP lipid-lowering adsorption therapy mainly applies hemoperfusion resins [68]. HP is straightforward and practical because it does not require a plasma separation step. High biocompatibility and low impact on blood cells make HP the preferred choice for lipid adsorption treatment [69]. Although HP can reduce lipid, amylase, and lipase levels during treatment, it cannot correct water, electrolyte, and acid-base balance [70]. Therefore, based on many studies, HP is recommended in combination with other BP techniques [71, 72]. A 2014 retrospective study reported that serum TG and cholesterol levels were significantly reduced in patients with HLSAP after HP treatment, with a 29.78% and 24.02% reduction in TG and cholesterol, respectively. Following hemofiltration treatment, HP resulted in a further rapid decline in TG, with serum TG levels decreasing by 49.02%, 62.81%, and 69.57% within three days of treatment [68]. However, monitoring and preventing coagulation complications during treatment is necessary because HP also adsorbs other blood components, such as platelets and coagulation factors [73]. In addition, because of the saturation of the adsorbent material, blood adsorption should be performed several times until the blood lipid level falls to normal.

### Continuous renal replacement therapy (CRRT)

CRRT utilized as a lipid-lowering treatment for HTG-AP relies on the adsorption of TG through a hemofilter. It simultaneously removes small molecules from the plasma via the diffusive convective effect of a semipermeable membrane. CRRT is a continuous, progressive, and hemodynamically more stable BP technique than PE. This tool can replace electrolytes in parallel with treatment, which is favorable for maintaining the water-electrolyte balance [74]. It is most often administered to patients who cannot tolerate intermittent BP or those who developed hypotension [75]. In addition, it is essential to note that the hemofiltration filter cannot clear blood lipids due to its small membrane area. Moreover, the hollow fibers of the filter are easily blocked by the TG. They are ineffective in removing large and medium molecular masses, resulting in the unsatisfactory removal of inflammatory mediators. Therefore, changing the blood filter several times is necessary to maintain its efficacy [68]. Unlike conventional therapies for renal failure, CRRT should be initiated within 48–72 h of onset for patients with HTG-AP [76–78]. Currently, CRRT treatment modalities for HTG-AP include continuous venovenous hemofiltration (CVVH) and high-volume hemofiltration (HVHF).

CVVH involves the use of a semipermeable membrane to filter out the ultrafiltrate by convection, supplemented with electrolyte solutions similar to plasma components. A 2019 retrospective study included 60 patients with SAP, 32 of whom received CVVH combined with conventional treatment (CVVH group), and 28 were treated with conventional treatment only (control group). The findings showed that APACHE II scores were significantly lower in the CVVH group than in the control group after seven days. CVVH therapy effectively decreased inflammatory mediators and improved biochemical and physiological parameters [78]. A randomized controlled study involving 37 patients showed that a high dose of CVVH improved hemodynamics and short-term survival to a greater extent than a low dose. Additionally, an intervention within 48 h was found to accrue more benefits than an intervention within 96 h [77].

The replacement fluid flow required by HVHF is more extensive than that required for ordinary hemofiltration. It enhances hemofiltration efficiency mainly by enhancing blood convection, with superior clearance of medium and small molecules compared to current renal replacement therapy. A randomized controlled study showed that HVHF was influential in rapidly clearing medium and large solutes such as TG within 9 h in the treatment group (HVHF group). However, the incidence of organ failure and hospital costs were significantly higher in the HVHF group than in the control group (insulin group), which had failed to improve the prognosis of patients with SAP [79]. Owing to the limited filtration capacity of HVHF for large and medium molecules, it is preferable to use HVHF in conjunction with other BP techniques. A retrospective controlled study in 2015 showed that after 48 h of high-volume hemofiltration combined with HP treatment, the APACHE II score, SOFA score, systolic blood pressure, diastolic blood pressure, heart rate, AML, and serum creatinine levels were significantly reduced in the treatment group than in the control group. Serum TG, cholesterol, and inflammatory mediators were significantly reduced, and the length of intensive care stay was shortened. However, no significant changes in serum cytokine levels have been observed in patients receiving conventional treatment alone [72]. Currently, the most available medical evidence supports the use of high doses [77]. Due to its high clearance efficiency, HVHF is regarded as a superior clinical treatment for HLSAP when combined with acute renal failure. HVHF efficiently removes circulating inflammatory mediators and toxins, making it an effective therapeutic option for managing HLSAP patients with concurrent renal complications.

Platelet activation plays a crucial role in the occurrence and development of AP [80]. The changes in AP with different causes are inconsistent [81–83]. Multiple studies have indicated that platelets directly participate in the systemic inflammatory process of AP [80, 81], and the key value of mean platelet volume (MPV) in determining the severity of AP when combined with other inflammatory markers and scoring systems has been discovered [81]. It is speculated that among various inflammatory mediators upregulated during non-hypertriglyceridemia-induced AP, IL-6 may be the primary factor leading to decreased MPV levels [84]. Conversely, pregnant women with hypertriglyceridemia-induced SAP exhibit higher MPV levels at onset, which aligns with elevated D-dimer levels, potentially increasing the propensity for thrombus formation and impairing pancreatic microcirculation [83]. CRRT can rapidly and effectively reduce D-dimer levels, improve pancreatic microcirculation, and correct hypercoagulability [85]. Therefore, monitoring MPV and D-dimer levels in AP patients undergoing CRRT can help assess the severity of HTG-AP. However, further large-scale prospective experiments are needed to validate these observations.

## Timing of application

Primary treatment strategies for HTG-AP are generally consistent with those for AP of other origins. However, many researchers emphasize the importance of studying specific etiologies and exploring gene therapy due to the unique nature of HTG-AP and its underlying causes. Patients with AP who undergo surgery in the acute phase have an increased risk of multiple organ dysfunction and death. Therefore, non-surgical treatment should be the principal treatment in the acute phase of HTG-AP. The primary goal of non-surgical HTG-AP treatment is to reduce serum TG levels as rapidly as possible within an adequate time frame [86]. Therefore, it is vital to determine the appropriate time to administer BP treatment to obtain a better prognosis.

Extensive studies have shown that serum TG levels decrease rapidly to < 5.65 mmol/L in patients with HTG-AP treated with BP within 24 h of diagnosis. Evidence suggests that BP treatment within 24 h of onset reduces serum TG by an average of 70% with the first attempt. However, it is not significant for preventing complications secondary to severe pancreatitis or reducing length of hospital stay [87]. Additionally, some reports suggest that the timing limitation of BP treatment can be extended to 48 h after disease onset. Several studies have reported that patients whose condition showed no improvement after conservative treatment demonstrated significant pain relief and general improvement within 48 h of receiving BP treatment [58, 79]. Therefore, it is now believed that early BP treatment within 24–48 h of HTG-AP diagnosis can help effectively reduce serum TG levels and improve patient prognosis. Early treatment with BP is more likely to result in a favorable prognosis [58].

CRRT is recommended within 48–72 h in the early stages of SAP to reduce inflammation and associated complications [88, 89]. Treatment may be initiated if accompanied by: (i) acute renal injury or urine output ≤ 0.5 mL/(kg·h); (ii) multiple organ dysfunction; (iii) early hyperthermia (> 39 °C) with tachycardia and dyspnea that does not improve significantly with conventional management; (iv) severe water-electrolyte and acid-base balance disturbances; (v) pancreatic encephalopathy with significant signs of toxicity; (vi) acute lung injury or acute respiratory distress syndrome (ARDS). In patients with HTG-AP, CRRT should be administered immediately after diagnosis to reduce serum TG levels and prevent SIRS [68]. Additionally, BP therapy can be initiated immediately in patients with severe vomiting who cannot tolerate oral lipid-lowering medications and have severe acute cardiac, hepatic, or renal organ failure [90].

Several studies have shown that a reduction in serum TG to < 5.65 mmol/L effectively halts the progression of HTG-AP [36, 37, 39, 90]. In patients who can tolerate oral dosing, it is necessary to consider initiating oral lipid-lowering therapy. Conversely, in patients with HTG-AP who develop organ dysfunction, lipid reduction cannot be used as a BP treatment endpoint [90, 91], and there is no supporting evidence for alternative indicators to serve as treatment endpoints for BP in such patients. Further research and clinical studies are needed to explore and establish the efficacy of alternative treatments in managing HTG-AP patients with organ dysfunction and high serum TG levels.

It should be noted that hemodialysis has relative contraindications in some cases: (i) severe bleeding tendency or internal bleeding (within 24 h of onset). (ii) Severe shock or hypotension (systolic < 80mmHg). (iii) Severe anemia (hemoglobin less than 30 g/L). (iv) Severe arrhythmia, cardiac insufficiency or coronary heart disease. (v) Gravity myopathy causes edema and heart force. (vi) Serious infections, such as sepsis. (vii) Advanced cancer.

## Conclusions and perspectives

HTG-AP is a form of pancreatitis caused by high TG levels. The disease progresses rapidly, resulting in severe disease with multiple organ dysfunction and poor prognosis. Reducing serum TG levels rapidly before the onset of severe complications and systemic inflammation is key to recovery and prevention of recurrence. The utilization of BP therapy for HTG-AP is now widely acknowledged. To reduce and further control serum TG levels below 5.65 mmol/L, physicians should consider personalized treatment and apply a combination of different modalities based on patient indications, which could help alleviate the condition, reduce associated complications, and prevent recurrence. Currently, the majority of available evidence is from retrospective, single-center studies with small sample sizes. Given that no expert consensus or clinical guidelines on the treatment of HTG-AP with BP is currently available, it is necessary to provide a reference for clinical decision-making based on more prospective, large-sample, multicenter studies.

## Data Availability

The raw data supporting the conclusions of this article will be made available by the authors, without undue reservation.
